# Impact of Resistant Maltodextrin Addition on the Physico-Chemical Properties in Pasteurised Orange Juice

**DOI:** 10.3390/foods9121832

**Published:** 2020-12-09

**Authors:** Elías Arilla, Marta Igual, Javier Martínez-Monzó, Pilar Codoñer-Franch, Purificación García-Segovia

**Affiliations:** 1 Food Investigation and Innovation Group, Food Technology Department, Universitat Politècnica de València, Camino de Vera s/n, 46022 Valencia, Spain; elarco@upv.es (E.A.); xmartine@tal.upv.es (J.M.-M.); pugarse@tal.upv.es (P.G.-S.); 2 Department of Pediatrics Obstetrics and Gynecology, University of Valencia, Avenida de Blasco Ibáñez, No. 15, 46010 Valencia, Spain; pilar.codoner@uv.es; 3 Department of Pediatrics, University Hospital Dr. Peset, Foundation for the Promotion of Health and Biomedical Research in the Valencian Region (FISABIO), Avenida Gaspar Aguilar, No. 90, 46017 Valencia, Spain

**Keywords:** prebiotic, resistant maltodextrin, orange juice, physico-chemical properties

## Abstract

Resistant maltodextrin (RMD) is a water-soluble fibre that can be fermented in the colon and exert prebiotic effects. Therefore, its addition to food and beverage products could be beneficial from both technological and nutritional viewpoints. However, to date, most studies have focused on the stability of the prebiotic fibre rather than its impact in the original food matrices. Therefore, this work aimed to evaluate the addition of RMD on the physico-chemical properties of pasteurised orange juice (with and without pulp). °Brix, pH, acidity, particle size distribution, density, turbidity, rheology, and colour were measured in orange juices with increasing RMD concentrations (2.5, 5, and 7.5%). Control samples without RMD were also prepared. RMD added soluble solids to the orange juice, affecting the °Brix, density, turbidity, and rheology. Slight colour differences were observed, and lower citric acid content was achieved because of orange juice replacement with RMD. Differences in particle size distribution were exclusively because of pulp content. Further studies are needed to elucidate if potential consumers will appreciate such physico-chemical changes in organoleptic terms.

## 1. Introduction

**Figure 1:**
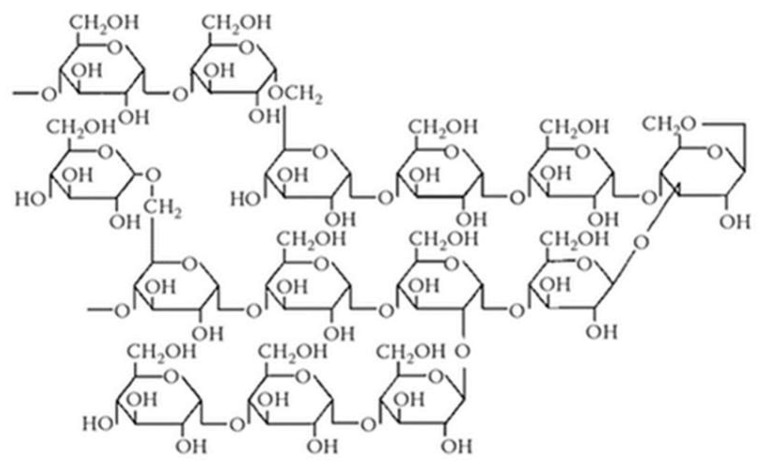
RMD chemical structure model.

Adding new food components to formulate novel nutritious and safe food products has become a method to improve the quality of human diets. One major food component that has tremendous interest from scientists, companies, and consumers is prebiotics fibres. Prebiotics are defined as “selectively fermented ingredients that allow specific changes, both in the composition and/or activity in the gastrointestinal microflora that confers benefits upon host wellbeing and health” [1]. The most studied and accepted prebiotic are non-digestible carbohydrates that include inulin, fructo-oligosaccharides, galacto-oligosaccharides, lactulose, and human milk oligosaccharides [2]. However, other food components, such as resistant maltodextrin (RMD), could exert functional effects too and, therefore, attract considerable interest. RMD is a water-soluble and fermentable fibre produced by the heat treatment of corn starch, indigestible in the small intestine but fermentable in the colon, resulting in enhanced short-chain fatty acid production [3]. Figure 1 shows the glycosidic linkages and molecular structure of RMD, which comprises a small ratio of saccharides with a degree of polymerisation (DP) 1–9 and many polysaccharides with a DP 10 or more. RMD contains a random distribution of 1–2 and 1–3 linkages, which are formed during the dextrinization process, and 1–4 and 1–6 linkages naturally found in starch [4]. In terms of food technology and food processing, RMD is a very user-friendly fibre because of its low viscosity and its high stability to heat and acid conditions. In addition, it is tasteless and flavourless, so it could be easily added to a wide range of food products.

In past studies, RMD has shown its potential prebiotic effect. In a double blind, randomised controlled crossover study, the daily intake of 25 g of RMD for 3 weeks (followed by a 2-week washout) increased faecal bifidobacteria counts and stool wet weight [5], suggesting health benefits. However, using RMD in food products is not limited to its potential prebiotic effect. For instance, in another double blind, randomised controlled crossover study, RMD demonstrated short-term decreased hunger and increased satiety hormones when ingested with a meal [6]. In addition, according to a systematic review of randomised placebo-controlled trials, the functional effect of RMD seems to be more effective in liquid foods rather than solid foods [7], so its use in beverages could be beneficial. These named studies suggest that RMD could have an adequate functional effect in a liquid matrix.

These functional effects can be linked to the maintenance of intestinal homeostasis because the gastrointestinal microflora plays a key role in the overall health status, affecting important human bodily functions such as metabolic, trophic, and protective [8]. Several factors have been shown to influence gastrointestinal microflora composition, being the environmental factors associated with diet and lifestyle the most predominant ones to shape it [9].

To provide a better diet, the food and beverage industry works to develop novel products that meet consumers’ requirements and population wellbeing. Therefore, the beverage industry, and especially the juice industry, is probably one of the most dynamic and innovative sectors. In accordance with the growing consumer inclination toward healthier food products [10], the beverage industry is increasing the number of healthier ingredients in their juices, for example, by developing functional beverages through adding prebiotics [11].

In terms of flavour, orange juice is the most demanded [11], as it is the most consumed fruit juice worldwide. Aspects like the fruit origin, fruit cultivar, maturity, juice processing conditions, packaging, and storage conditions affect the physico-chemical properties of the juice. Moreover, orange juice, one of the most representative citrus juices, contains many nutritive and biologically active micronutrients, besides its natural sugar content. The most significant micronutrients for orange juice are potassium, copper, folate, vitamin C, flavonoids (mostly hesperidin), and dietary fibres [12]. From the viewpoint of juice processing, one of the primary considerations is the technology applied to assure the microbial stabilisation. Although alternative processing treatments have been developed (for example, high-pressure processing technologies or pulsed electric fields), thermal pasteurisation is still the most cost-effective method to reduce microbial populations and enzyme activity [13].

Because of their technological characteristics, food components perceived as prebiotics have shown not only an upgrade in the nutritious quality of the food product but also an improvement in the quality regarding sensory properties, texture, and physico-chemical properties [14]. Thus, based on previous evidence, adding RDM could be beneficial from nutritional and technological viewpoints, to give a novel potentially prebiotic pasteurised orange juice. However, it is necessary to specifically evaluate how each functional food component affects the matrix in which it is added. In addition, most of the studies that have been previously completed in this field focused on the stability of the prebiotic fibre in fruit juice processing and storage conditions or in the functionality of the finished beverages [15,16,17,18]. These are undoubtedly of great importance, but few studies show how the prebiotic fibres added affect the physico-chemical properties of the finished beverage. In addition, most of the studies that have been done were focused on the effect of prebiotic addition on stability, storage conditions, or functionality of the prebiotic fibre in the finished beverages [15,16,17,18]. Only a few studies show how the addition of prebiotic fibres impact the physico-chemical properties of the finished beverage. Therefore, this study aimed to evaluate the addition of RMD on the physico-chemical properties of pasteurised orange juice (with and without pulp). How the RMD affects °Brix, pH, acidity, particle size distribution, density, turbidity, rheology, and colour were elucidated to help develop novel functional products that could be accepted by a consumer.

## 2. Materials and Methods

### 2.1. Raw Materials

This study was conducted with freshly squeezed orange juice supplied by Refresco Iberia S.A.U. (Valencia, Spain). All oranges were from Spanish origin. RMD (Fibersol-2) added to the juice was purchased from ADM/Matsutani, LLC (Decatur, IL, USA). Frozen pasteurised orange pulp was provided by a local fruit processing company (Zumos Valencianos del Mediterráneo, Valencia, Spain).

### 2.2. Sample Preparation and Pasteurisation

A total of eight samples of orange juice were prepared. Four were orange juice with pulp (OJP) and the other four were orange juice without pulp (OJWP). Fresh orange juice was directly collected from the industrial squeezed lines. Orange pulp (2.5%) was added to the OJP samples, and increasing RMD concentrations (2.5, 5, and 7.5%) were added into both OJP and OJWP samples. Both orange pulp and RMD concentrations were applied in weight/weight percentage. Pulp content was homogenised using a stirrer (LH Overhead Stirrer, VELP Scientifica, Usmate, Italy), by applying 200 rpm for 5 min. Increasing RMD concentrations were mixed and stirred (200 rpm, 15 min) into both OJP and OJWP samples. Thus, for a finished beverage portion of 200 g, either 5, 10, or 15 g of RMD would be ingested, enough to display functional effects according to other studies [5,6,7]. Control samples without RMD addition (OJP0 and OJWP0) were also prepared, and they complied with the European Fruit Juice Association (AIJN) orange juice guidelines [19], so no adulteration or deviation occurred during the juice extraction. Finally, all samples were pasteurised (Fruchtsaftdispenser, Mabo Steuerungselemente GmbH, Eppingen, Germany) at 85 °C for 10 s, and were hot filled into 250 mL polyethylene terephthalate (PET) bottles. All bottles were immersed in a cold-water bath (<10 °C) for 30 min to cool down their temperature after the heat treatment.

### 2.3. Physico-Chemical Determinations

#### 2.3.1. °Brix, Acidity, and pH

Measurement of total soluble solids (°Brix) was conducted using refractometry (Abbemat 200, Anton Paar, Graz, Austria). Acidity, expressed as grams of citric acid per 100 mL of orange juice (gCA/100 mL), was determined using a DL53 acid titrator (Mettler Toledo, Greifensee, Switzerland). Determination of pH was made using a Basic 20 pH meter (Crison, Alella, Spain). All determinations were performed in triplicate in accordance with AOAC guidelines [20].

#### 2.3.2. Particle Size Distribution

Juice particle size distribution was determined by applying the laser diffraction method and Mie theory, following the ISO13320 regulation [21], by using a particle size analyser (Malvern Instruments Ltd., Mastersizer 2000, Malvern, UK) equipped with a wet sample dispersion unit (Malvern Instruments Ltd., Hydro 2000 MU, Malvern, UK). Laser diffraction reports the volume of material of a given size, since the light energy reported by the detector system is proportional to the volume of material present. The Mie theory requires the information on both the sample and the dispersant optical property. For orange juice, the particle refraction and absorption were 1.52 and 0.1, respectively, and the water refraction index was 1.33. The sample was dispersed in distilled water and pumped through the optical cell under moderate stirring (1800 rpm) at 20 °C. The volume (%) against particle size (in µm) was obtained and the size distribution was characterised by the volume mean diameter (*D* (4,3)). The standard percentile *d* (0.1) or size of particle below which 10% of the sample lies and *d* (0.9) or size of particle below which 90% of the sample lies were also considered for juice characterisation.

#### 2.3.3. Rheological Measurements

Juice flow behaviour was analysed using a controlled shear stress rheometer coupled to a thermostatic bath (Thermo Electron Co., Haake RheoStress 1, Waltham, MA, USA) with coaxial cylinders (Z34 DIN) using sensor system set at 20 °C following the Igual et al., [22] methodology. A relax time of 900 s was selected for the sample before running the test. Shear rate, (*γ*; s^−1^), was increased from 0 to 150 s^−1^ in 20 step (fixed duration for each step 30 s) and shear stress *σ* (Pa), was recorded.

#### 2.3.4. Density

Density was determined by using a pycnometer (50 mL) and distilled water at 25 °C as a reference.

#### 2.3.5. Turbidity

Orange juice was centrifuged at 3000 rpm for 10 min. The turbidity of the upper layer solution was determined using a spectrophotometer UV-VIS (Thermo Scientific, Helios Zeta UV-Vis, Loughborough, LE, UK) at 600 nm, as described by Chandler and Robertson [23]. Sample transmittance (T) was obtained in relation to distilled water, and the turbidity (Tb) was calculated using Equation (1) [24].

(1) Tb (%)=100−T

#### 2.3.6. Colour Measurement

Colour values were obtained from the reflection spectrum. Samples colour was measured using a colorimeter (CM-700d, Konica Minolta, Tokyo, Japan) with a standard illuminant D65 and a visual angle of 10°. Results were obtained in terms of L* (brightness: L* = 0 (black), L* = 100 (white)), a* (−a* = greenness, +a* = redness), and b* (−b* = blueness, +b* = yellowness), according to the CIELab system [25]. Chroma, C*ab (saturation) and hue angle, h*ab were also calculated, using equations 2 and 3, respectively. The colour difference was calculated regarding the control samples in each case, in all OJP and OJWP samples, to evaluate the RMD addition effect.

(2) $$C_{\mathrm{ab}}^{*}={\left(\left(a^{*2}+b^{*2}\right)\right)}^{1/2}$$

(3) $$h_{\mathrm{ab}}^{*}=\arctan\left(\frac{b^{*}}{a^{*}}\right)$$

### 2.4. Statistical Analysis

Analysis of variance (ANOVA) was applied with a confidence level of 95% (*p <* 0.05), to evaluate the differences among samples. Furthermore, a correlation analysis among studied properties of juices and RMD concentration was made, with a 95% significance level. Statgraphics (Centurion XVII Software, version 17.2.04, Statgraphics Technologies, Inc. The Plains, VA, USA) was used.

## 3. Results and Discussions

°Brix, acidity, and pH were evaluated as the basic control parameters, as is the general protocol in the juice industry (Table 1). Increasing concentrations of RMD implied a significant increase in total soluble solids in both OJP and OJWP samples (*p <* 0.05). This makes sense since, as explained, RMD is a water-soluble fibre. OJWP samples showed slightly higher values of °Brix (*p <* 0.05), mainly because a small percentage of orange juice was replaced in OJP samples by orange pulp, which is an insoluble fibre. Ghavidel et al. [17] also reported than an increase in fibre content (fructo-oligosaccharides, FOS) produced an increase in the total soluble solids in an orange juice-based sugar-added beverage. In addition, the substitution of sugar by other prebiotic fibres, namely oligofructose and inulin, did not change the °Brix range of papaya nectar [26]. Because of this soluble solid addition to the matrix and its light sweetness taste, such fibres have been proposed as sugar replacers, among other food technology applications [27,28]. This could be beneficial in acidic food products, such as orange juice, to help balance its sensory profile without adding sugar but adding functional ingredients.

However, higher RMD concentrations significantly decreased acidity values in both OJP and OJWP samples (*p <* 0.05). This is because RMD addition helped to reduce the quantity of raw orange juice, and therefore citric acid. Moreover, OJP samples had significantly higher acidity values than OJWP (*p <* 0.05). In terms of pH, the differences were small but significant (*p <* 0.05), as OJP samples presented lower pH values than OJWP. Therefore, orange pulp addition showed an impact on the acidity and pH values (*p <* 0.05), whereas RMD addition decreased citric acid content by replacing orange juice content (*p <* 0.05). The acidity of the orange pulp could be higher than the acidity from the orange juice, thus leading to an increase in acidity and a decrease in pH in OJP samples. RMD addition had less impact on the pH.

°Brix, pH, and acidity values of the control samples are hard to compare with those in other studies, since the oranges used for the juice extraction were all from Spanish origin. Citric acid values reported from Mexican orange juices were lower [29]. However, °Brix and pH were almost the same as those reported from Cortés et al. [30], who used the Navel cultivar from Spain. This enhances the importance of the raw material origin and the complexity to properly compare the physico-chemical properties in fruit-derived products.

**Figure 2:**
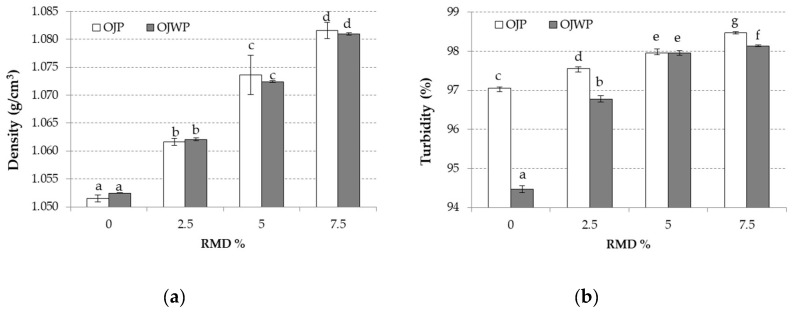
(**a**) Mean values (and standard deviations) of density of pasteurised orange juice; (**b**) Mean values (and standard deviations) of turbidity of pasteurised orange juice. Letters indicate homogeneous groups established by the ANOVA (*p <* 0.05) for each parameter analysed. OJP, orange juice with pulp; OJWP, orange juice without pulp.

Juice density is also an important quality control parameter in the juice industry [31]. Figure 2a shows that density values were not affected by pulp addition (*p >* 0.05) and that they increased as RMD concentration increased (*p <* 0.05). This could be explained because RMD was dissolved completely in the orange juice. Moreover, it is widely known that, in fruit juices, the soluble solids quantity affects density values, while insoluble solids, such as cloud and pulp, contribute little to density measurements [31]. The relationship between density and soluble solids has been extensively studied and regression models have been developed, like the one obtained by Ramos and Ibarz [32].

Turbidity is represented in Figure 2b. Turbidity provides a measure of the concentration of disperse particles in a solution by measuring its light-scattering properties [33]. Therefore, as RMD was completely dissolved, higher RMD concentrations led to higher turbidity values (*p <* 0.05). However, in contrast to the density measurements, pulp content played a role in the turbidity values (*p <* 0.05), clouding OJP samples. This can be checked by comparing turbidity values of the control samples (OJP0 and OJWP0). However, the clouding effect because of pulp content was limited as turbidity difference between OJP and OJWP samples decreased as RMD concentration raised. For example, no turbidity difference was found in both 5% RMD-added samples.

**Figure 3:**
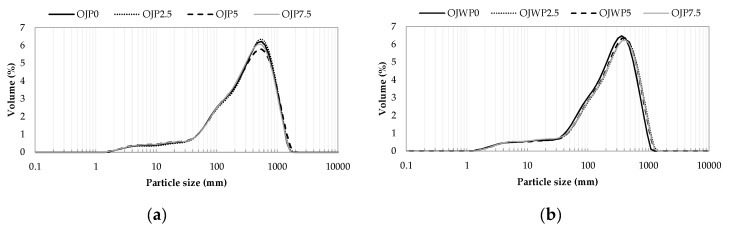
(**a**) Volume particle size distributions (representative curves) of pasteurised orange juice with pulp; (**b**) Volume particle size distributions (representative curves) of pasteurised orange juice without pulp. OJP, orange juice with pulp; OJWP, orange juice without pulp.

Volume particle size distribution is represented for the OJP samples (Figure 3a) and OJWP samples (Figure 3b). Both OJP and OJWP samples showed a similar trend, as it can be observed in Figure 3. Table 2 compiles the mean values (and standard deviations) of volume mean diameter *D* (4,3) and the standard percentiles *d* (0.1), *d* (0.5), and *d* (0.9). Particles size of OJP samples presented significantly greater volume mean diameter than OJWP samples (*p <* 0.05). RMD addition did not have an impact in the volume mean diameter (*p >* 0.05), as it was dissolved in the orange juice. Therefore, the difference between OJP and OJWP samples regarding volume mean diameter were exclusively because of pulp content (*p <* 0.05). This phenomenon is demonstrated by comparing the standard percentiles. As the number of analysed particles grows, the particle size distribution becomes homogenised; this is seen in Table 2 by comparing *d* (0.1) values to *d* (0.9) values. In the first percentile d (0.1), greater differences in particle size distributions were found between all orange juice samples (*p <* 0.05). However, by increasing the number of particles analysed (*d* (0.9)), the same relationship is obtained as with the volume mean diameter. Thus, OJP obtained a greater quantity of particles of larger size (*p <* 0.05). The particle size distribution obtained for these orange juice samples differed from the one performed by Stinco et al. [34], who, on average, reported larger particle size for the industrially squeezed orange juices (both fresh and pasteurised). Achieving smaller particles could be beneficial as the food matrix is one of the key factors related to the release of bioactive compounds, such as carotenoids [34].

**Figure 4:**
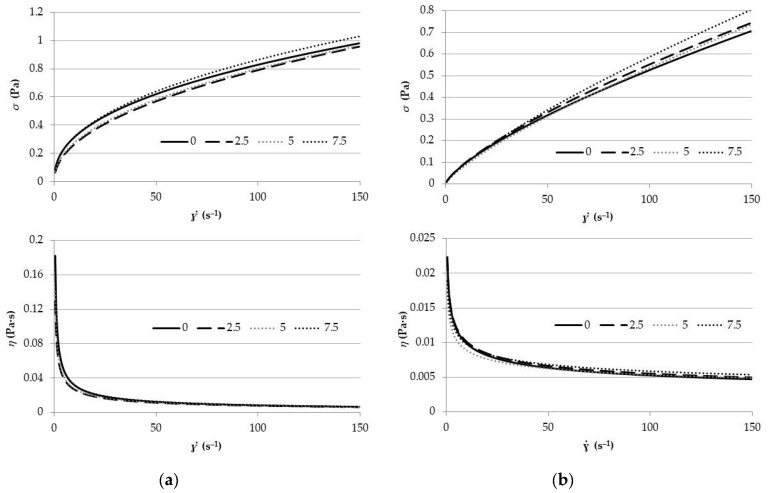
(**a**) Flow behaviour (*σ*-*γ*) and viscosity profiles (*η*-*γ*) of pasteurised orange juice with pulp (OJP); (**b**) Flow behaviour (*σ*-*γ*) and viscosity profiles (*η*-*γ*) of pasteurised orange juice without pulp (OJWP).

Regarding rheology, all orange juices showed a non-Newtonian, non-time dependent, pseudoplastic behaviour as observed in Figure 4. The obtained flow curves were well-fitted (*R*^2^ ≥ 0.99) to the Ostwald de Waele model (Equation (4)), where *k* is the consistency index (Pa·s^n^) and *n* is the flow index (Table 3). This mathematical relationship is useful because of its simplicity [35].

(4) $$\sigma =k\;{\dot{\gamma }\;}^{n}$$

OJP samples showed significantly higher *k* values than OJWP samples (*p <* 0.05), meaning that pulp addition could slightly increase the viscosity of the orange juice. This can be observed in Figure 4 comparing viscosity profiles of OJP and OJWP. Rega et al. [36] also demonstrated that viscosity increased as pulp content increased. This could be explained because suspended solids increases the apparent viscosity of the fruit juice [37], probably because of the pectin content of orange pulp [38]. RMD addition did not produce a significant change in *k* of OJWP samples (*p >* 0.05). Moreover, RMD addition did not produce a clear trend in the *k* of both OJP and OJWP samples. Despite soluble solids supposedly increasing viscosity [37], in this study, RMD addition did not show differences in the viscosity profiles. However, OJWP samples marked significantly higher *n* values than OJP samples (*p <* 0.05), thus, its flow behaviour was slightly more Newtonian than OPJ samples. This suggests that OJP exhibits a more pseudoplastic behaviour than OJWP (Figure 4). RMD addition had also a significant effect on the *n* behaviour in OJWP, as *n* values in samples with 5 and 7.5% RMD increased (*p <* 0.05). However, orange pulp addition seems to have a stronger impact on the rheology of orange juice samples.

A statistical correlation was conducted to explain the relationship between the studied parameters in the orange juice samples. There were significant correlations among particle size parameters and rheological values according to Pearson coefficients (*p <* 0.05). As was observed in other citric fruit juices [22], *k* showed a positive correlation with *D* (4,3) (0.9238), *d* (0.1) (0.8512), *d* (0.5) (0.9068), and *d* (0.9) (0.9263); *n* showed a negative correlation with *D* (4,3) (−0.9609), *d* (0.1) (−0.8932), *d* (0.5) (−0.9467), and *d* (0.9) (−0.9609). Therefore, the higher the particle size in juices, the higher the consistency index in juices but the lower the flow index. Moreover, *k* and *n* were correlated with pH and acidity showing −0.7875 and 0.8766, and 0.7628 and −0.9168 Pearson coefficients, respectively.

**Figure 5:**
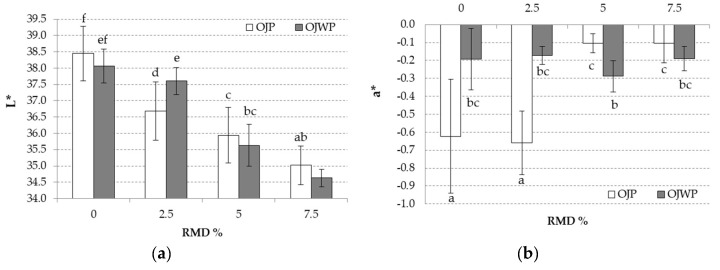

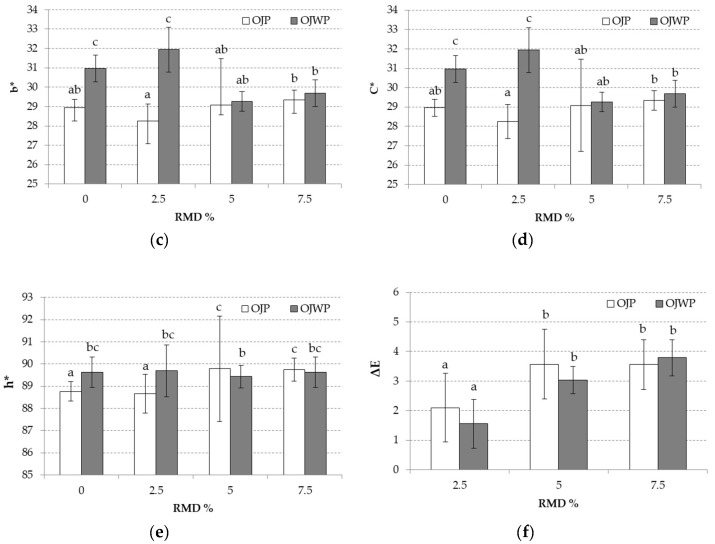
Mean values (and standard deviations) of colour coordinates L* (**a**), a* (**b**), b* (**c**), C* (**d**), and h* (**e**) and total colour differences (ΔE, (**f**)) of pasteurised orange juice. Letters indicate homogeneous groups established by the ANOVA (*p <* 0.05) for each parameter analysed. OJP, orange juice with pulp; OJWP, orange juice without pulp.

Finally, colour is one of the most important parameters for consumers, as it is related to quality perception [30]; colour changes are shown in Figure 5. It was shown that higher RMD concentrations significantly decreased L* values (*p <* 0.05) (Figure 5a), thus that all orange juices turned slightly darker, as RMD concentration was raised. This could be explained as higher RMD concentrations helped to reduce orange juice content. Orange pulp showed no significant effect on the L* values (*p >* 0.05). L* presented significant correlations with density (0.9493) and turbidity (0.7467) (*p <* 0.05). Igual et al. [22] also found a high correlation between L* and turbidity in grapefruit juices. In addition, OJP0 and OJP2.5 had the lowest a* values (*p <* 0.05) (Figure 5b), which implies that they were greener than the rest. This could probably be explained because RMD addition led to a protective effect on the carotenoids content [39], therefore leading to a protective effect in the reddish tones during the pasteurisation process. For the yellow-blue content (b*, Figure 5c), RMD addition showed no significant effect (*p >* 0.05), while orange pulp content had a significant effect (*p <* 0.05) on b* values. Thus, in the OJWP samples, an increasing addition of RMD seemed to lower the yellowish tones, as OJWP5 and OJWP7.5 had lower b* values. However, few differences exist in the OJP because of RMD addition. This suggests that orange pulp content could have a protective effect on the natural yellow/orange tones of orange juice. C* values (Figure 5d) resulting from Equation (2) were almost the same as b*, as a* values were all close to 0. Regarding h*, RMD addition on OJWP samples did not play a significant role (*p >* 0.05). However, h* values (Figure 5e) were significantly increased (*p <* 0.05) as RMD concentration was higher in OJWP samples.

RMD addition in OJP and OJWP samples had almost the same result in terms of total colour difference (ΔE) regarding control samples (Figure 5f), thus, that pulp content did not interfere in colour differences (*p >* 0.05). Total colour differences between juices with RMD 2.5% and the control samples were lower than three units. Therefore, they are not perceptible to human eye, which only distinguishes colour differences if ∆E is larger than three [40]. Increasing RMD addition implied small but significant (*p <* 0.05) colour differences, but they were limited because samples with 5% and 7.5% RMD had almost no colour difference between them.

## 4. Conclusions

In this study, physico-chemical differences were found due to adding RMD to orange juice with and without pulp. Samples with 7.5% added RMD presented a greater impact on the physico-chemical properties in both OJP and OJWP samples. With RMD, a soluble-water fibre, its addition to orange juice increased the total soluble solids content, which raised Brix, density, and turbidity values, the last more evident in OJWP samples. Citric acid content was lowered because of the orange juice replacement by RMD, and small but significant changes were observed in terms of pH. Differences in particle size distribution were exclusively because of pulp content. Orange pulp content, and not RMD addition, appears to have an impact on the orange juice rheology. Slight colour differences were found; however, only higher RMD concentration would be perceptible by the human eye.

This study demonstrates that RMD addition in a wide range of concentrations is feasible from a food technology viewpoint. However, the optimal dose of RMD will depend on the functional effect to be achieved.

## Figures and Tables

**Table 1:** Mean values (and standard deviations) of °Brix, pH, and acidity of pasteurised orange juice.

Sample	°Brix	Acidity (g CA/100 mL)	pH
OJP0	11.36 (0.04) ^a^	0.773 (0.003) ^h^	3.36 (0.04) ^a^
OJP2.5	13.58 (0.03) ^c^	0.756 (0.003) ^g^	3.46 (0.08) ^b^
OJP5	15.83 (0.08) ^e^	0.733 (0.002) ^f^	3.50 (0.07) ^bc^
OJP7.5	17.98 (0.04) ^g^	0.7133 (0.0006) ^e^	3.44 (0.04) ^b^
OJWP0	11.52 (0.05) ^b^	0.686 (0.003) ^d^	3.55 (0.03) ^cd^
OJWP2.5	13.75 (0.03) ^d^	0.671 (0.002) ^c^	3.59 (0.03) ^d^
OJWP5	15.96 (0.04) ^f^	0.656 (0.002) ^b^	3.54 (0.03) ^cd^
OJWP7.5	18.18 (0.08) ^h^	0.6380 (0.0005) ^a^	3.57 (0.03) ^cd^

The same letter in superscript within column indicates homogeneous groups established by ANOVA (*p <* 0.05). OJP, orange juice with pulp; OJWP, orange juice without pulp.

**Table 2:** Mean values (and standard deviations) of volume mean diameter (μm) *D* (4,3), standard percentiles (μm) *d* (0.1), *d* (0.5), and *d* (0.9) of pasteurised orange juice.

Sample	*D* (4,3)	*d* (0.1)	*d* (0.5)	*d* (0.9)
OJP0	368 (27) ^b^	44 (4) ^d^	305 (25) ^cd^	791 (55) ^b^
OJP2.5	382 (33) ^b^	49 (6) ^e^	321 (30) ^d^	814 (71) ^b^
OJP5	374 (39) ^b^	38 (5)^c^	299 (30) ^b^	825 (92) ^b^
OJP7.5	361 (27) ^b^	40 (4) ^c^	296 (22) ^b^	783 (61) ^b^
OJWP0	300 (14) ^a^	33 (3) ^b^	253 (12) ^a^	637 (33) ^a^
OJWP2.5	297 (20) ^a^	28 (3) ^a^	248 (16) ^a^	638 (44) ^a^
OJWP5	288 (12) ^a^	30 (2) ^ab^	241 (10) ^a^	615 (28) ^a^
OJWP7.5	289 (15) ^a^	27 (3) ^a^	240 (13) ^a^	623 (32) ^a^

The same letter in superscript within column indicates homogeneous groups established by ANOVA (*p <* 0.05). OJP, orange juice with pulp; OJWP, orange juice without pulp.

**Table 3:** Mean values (and standard deviations) of consistency index (*k*), flow index (*n*) of pasteurised orange juice.

Sample	*k* (Pa·s*^n^*)	*n*
OJP0	0.121 (0.009) ^c^	0.4276 (0.0102) ^a^
OJP2.5	0.0891 (0.0009) ^b^	0.47 (0.02) ^c^
OJP5	0.096 (0.005) ^b^	0.461 (0.012) ^bc^
OJP7.5	0.117 (0.015) ^c^	0.43 (0.02) ^ab^
OJWP0	0.0184 (0.0002) ^a^	0.728 (0.002) ^d^
OJWP2.5	0.0186 (0.0002) ^a^	0.73675 (0.00106) ^d^
OJWP5	0.01477 (0.00009) ^a^	0.7800 (0.0012) ^e^
OJWP7.5	0.01618 (0.00006) ^a^	0.7799 (0.0013) ^e^

The same letter in superscript within column indicates homogeneous groups established by ANOVA (*p <* 0.05). OJP, orange juice with pulp; OJWP, orange juice without pulp.
